# Effects of Mixing Ratios on Branch Development in Young Mixed Plantations of *Betula alnoides* and *Castanopsis hystrix*

**DOI:** 10.3390/plants14243730

**Published:** 2025-12-06

**Authors:** Yangdong Zou, Chunsheng Wang, Yuhan Chang, Haifeng Yin, Qiong Dong, Jie Zeng

**Affiliations:** 1 Research Institute of Tropical Forestry, Chinese Academy of Forestry, Guangzhou 510520, China; cool789123456@163.com (Y.Z.); changyh00@163.com (Y.C.); yhfeng312@163.com (H.Y.); zengj69@caf.ac.cn (J.Z.); 2 Hunan Botanical Garden, Changsha 410116, China; 3 College of Forestry, Southwest Forestry University, Kunming 650224, China; dqyeam@swfu.edu.cn

**Keywords:** branchiness, mixing proportion, branch metrics, morphological variation, crown architecture

## Abstract

Branch characteristics (quantity, morphology, and distribution) are critical determinants of tree growth and wood quality. However, the influence of species mixing, particularly mixing ratios, on branch development remains poorly understood. This study examined the branch attributes of *Betula alnoides* and *Castanopsis hystrix* in a six-year-old mixed-species trial plantation including monoculture of each species, and three mixtures at ratios of 1:1, 1:3, and 1:5 (*B. alnoides*–*C. hystrix*) in Pingxiang, Guangxi, China. Branch quantity (number, proportion, and density), morphology (diameter, length, and angle), and distribution (vertical and horizontal) were measured or recorded from 40 sampled dominant or codominant trees (20 *B. alnoides* and 20 *C. hystrix*). The results showed that mixing significantly increased the number and density of branches over 124.2% and 53.2%, respectively, in the lower crown (below 10 m) of *B. alnoides*, with these metrics positively correlated to the proportion of *C. hystrix,* while mixing exerted limited effects on branch quantity and size of *C. hystrix*. The 1:3 and 1:5 mixtures yielded more small branches (diameter < 10 mm) as well as more large branches (>25 mm) for *B. alnoides.* Branch distribution was almost uniform in different horizontal directions for both species, while variations in branch quantity and morphology along the stem were primarily species-specific; and both aspects remained consistent across the different mixing ratios. In conclusion, mixing *B. alnoides* with a low proportion of *C. hystrix* is proposed to produce high-quality solid wood for both species. Future studies should investigate alternative mixing patterns and higher proportions of *B. alnoides* in mixture with *C. hystrix* to optimize large-size and high-quality timber production.

## 1. Introduction

Branch attributes, including number, size, and distribution, are crucial determinants of canopy structure, stand dynamics, and stand productivity, thereby influencing ecosystem characterization and functioning [1,2]. Furthermore, branches significantly impact commercial timber quality. The dead knots that develop after branch shedding can adversely affect wood texture and appearance, alter physical and mechanical properties [3,4], and result in the downgrading of timber logs or sawn boards [5,6]. Although studies on branch development over recent decades have predominantly focused on planting density and artificial pruning in monocultures [7,8], the effects of species mixing on branch development remain relatively unexplored.

Compared to monocultures, mixed forests generally provide multiple advantages, including increased stand productivity [9,10], reduced pest and disease incidence [11], improved soil nutrient status [12], and greater stand stability [13,14]. Consequently, many countries have increasingly shifted from monocultures to mixed forests in recent decades [4,15]. Although knowledge regarding tree growth, stand productivity, nutrient cycling and other ecosystem functions in mixed-species forests has advanced considerably, the effects of species mixing, particularly mixing ratio, on branch development remain poorly understood [16]. There is a clear need for more intense investigation into the influence of mixed-species neighborhoods on branch development.

In mixed-species plantations, increased structural heterogeneity and species complementation can enhance resource use efficiency and mitigate competition, potentially promoting tree growth [17], and modulating branch quality and morphology [16,18,19]. Moreover, neighboring trees may act as “trainer trees”, accelerating natural pruning in the lower and most valuable stem sections of crop trees [20]. In essence, the impact of species mixing on branch development is likely contingent upon species interactions and competitive capacity, which are intrinsically linked to the mixing ratio within the stand.

*Betula alnoides* Buch.-Ham. ex D. Don is a valuable deciduous, shade-intolerant tree species native to Southeast Asia and south China. Known for its fast growth and adaptability to a wide range of soils, altitudes, and climatic conditions, it can achieve an average annual increment in diameter at breast height and tree height of up to 2.0 cm and 2.0 m, respectively, in young plantations. Its wood, resistant to warping and cracking, is commonly used for flooring, furniture, interior decoration, and veneer [21,22,23], making it well-suited for large-sized timber production on a relatively short rotation (25–30 years). Additionally, *B. alnoides* is also an environmentally favorable tree species, permitting adequate light penetration. The planting area of *B. alnoides* in southern China exceeds 20,000 hectares. *Castanopsis hystrix* Miq., an important broad-leaved species in the same region, exhibits greater shade tolerance and a slower growth rate than *B. alnoides*, especially in young stages. It is typically managed on a rotation of 30–35 years [24]. Although both species were historically predominantly managed in monoculture in southern China, significant limitations emerged during their cultivation. Given their complementary growth attributes and adaptability, large-scale, even-aged, or uneven-aged mixed forests with different mixing ratios of the two species have been established.

We hypothesized that mixing *B. alnoides* with *C. hystrix* could effectively control branch development for both species, and from a branch development perspective, a higher proportion of *C. hystrix* is suboptimal for their mixture. This study specifically aims to (1) quantify differences in branch quantity, morphology, and distribution for both species between monoculture and mixture, as well as across mixtures with varying ratios; and (2) provide evidence-based recommendations for establishing and managing mixed forests of the two species.

## 2. Results

### 2.1. Branch Quantity

Mixing with *Castanopsis hystrix* significantly increased the number of branches in the crown section below 10 m for *Betula alnoides* compared to its monoculture (Table 1), particularly in the 1:3 and 1:5 mixtures (*B. alnoides*–*C. hystrix*). The highest branch number was observed in the 1:3 mixture. In contrast, in the crown section above 10 m, branch number was markedly higher in monoculture of *B. alnoides* than in the 1:3 mixture (Table 1). No dead branches were recorded in either crown section of *B. alnoides*. Similarly, branch density in the crown section below 10 m was significantly higher in mixtures than in monoculture, also peaking in the 1:3 mixture. However, significant differences in branch density were absent in the crown section above 10 m across all treatments (Table 1).

For *C. hystrix*, the total branch number was markedly higher in monoculture than in mixtures with *B. alnoides*, although no significant differences were detected in both live and dead branch numbers among any of the treatments (Table 1). Likewise, the proportion and density of live or dead branches, as well as total branch density, showed no significant differences across all treatments.

### 2.2. Branch Morphology

Branch angle, length, and diameter exhibited no significant differences across mixing ratio treatments for either *B. alnoides* or *C. hystrix* (Table 2). However, these traits were greatly influenced by crown layer. Branch orientation significantly affected only the branch diameter and length of *B. alnoides*. Significant two-way interactions were identified: mixing ratio × crown layer affected branch angle and length in both species; mixing ratio × branch orientation influenced branch length of *B. alnoides* and branch angle of *C. hystrix*; and crown layer × branch orientation impacted branch length of *B. alnoides*. Additionally, the three-way interaction of mixing ratio × crown layer × branch orientation significantly affected the branch diameter and length of *B. alnoides* and branch angle of *C. hystrix*.

For *B. alnoides* branches in the crown section below 10 m, significant differences in branch diameter and length were absent among monoculture and mixtures with different mixing ratios (Table 3). However, branch angle was larger in mixtures than in monoculture, with the difference reaching significance in the 1:1 mixture. In the crown section above 10 m, branches were larger in monoculture than in mixtures, with significant difference in diameter and length between the monoculture and the 1:3 mixture. The diameter of the largest branch increased as the proportion of *B. alnoides* decreased, with a significant difference observed only between the monoculture and the 1:5 mixture. In contrast, the height of the largest branch did not differ significantly across all treatments. For *C. hystrix*, branch morphology was not significantly affected by mixing treatments (Table 3).

**Figure 1:**
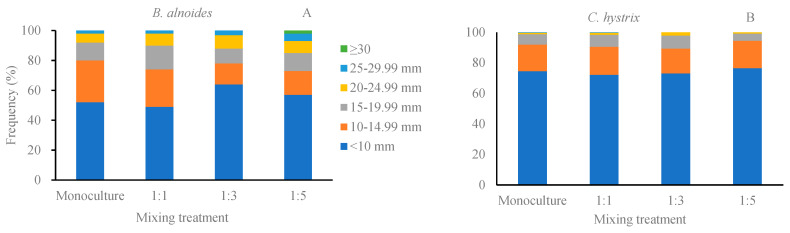
Frequency distribution of branch diameter for *Betula alnoides* and *Castanosis hystrix* in their monocultures and mixtures with different ratios. (**A**) *B. alnoides*; (**B**) *C. hystrix*.

Compared to monoculture, *B. alnoides* in the 1:3 mixture exhibited a higher frequency of the smallest diameter class (<10 mm) and a lower frequency of relatively low-diameter class branches (10–14.99 mm and 15–19.99 mm), with the proportion of these diameter classes increasing or decreasing by more than 10.0%. Furthermore, no branches exceeding 30 mm in diameter were observed in the monoculture, 1:3 and 1:1 treatments, whereas the 1:5 treatment produced more large branches (≥30 mm diameter) than other treatments (Figure 1A). For *C. hystrix*, mixing with *B. alnoides* did not significantly affect branch diameter frequency distribution. It is noteworthy, however, that branches larger than 25 mm in diameter were absent in both the 1:3 and 1:5 treatments (Figure 1B).

### 2.3. Branch Spatial Distribution

**Figure 2:**
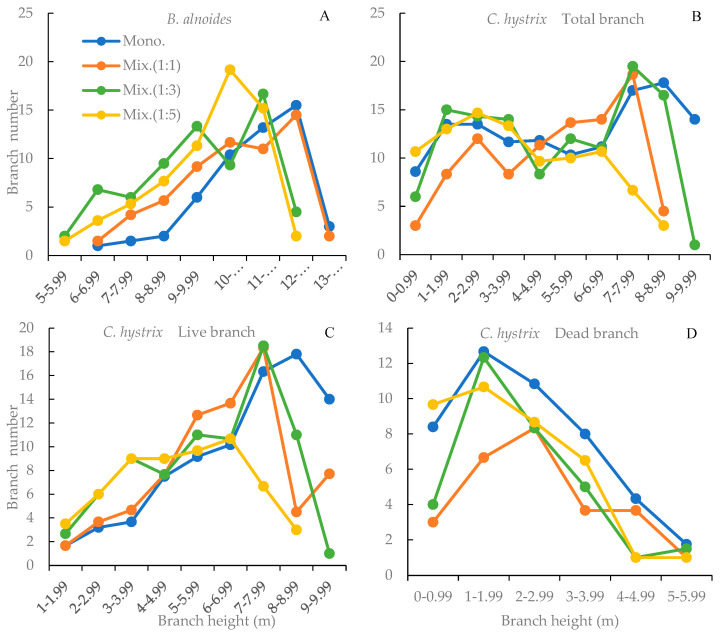
Vertical distribution of branch quantity for *Betula alnoides* and *Castanosis hystrix* in their monocultures and mixtures with different ratios. (**A**) *B. alnoides*; (**B**) Total branches of *C. hystrix*; (**C**) Live branches of *C. hystrix*; (**D**) Dead branches of *C. hystrix*.

For *B. alnoides*, branch number initially increased and then declined with ascending crown layer, peaking at 10–10.99 m in the 1:5 treatment, 11–11.99 m in the 1:3 treatment, and 12–12.99 m in the 1:1 treatment and the monoculture, indicating that a higher proportion of *C. hystrix* shifted the crown layer with peak branch number downward (Figure 2A). For *C. hystrix*, branch number was generally low in the middle crown layers (4–6.99 m) across all treatments except the 1:1 mixture, without regard to the lowest and top crown layers (Figure 2B). The number of live branches increased from 1–1.99 m to 7–7.99 m crown layers (Figure 2C), while the number of dead branches decreased from 1–1.99 m to 5–5.99 m crown layers. Moreover, each crown layer contained considerably more dead branches in monoculture than in mixtures, except for the 0–0.99 m crown layer in the 1:5 mixture (Figure 2D).

**Figure 3:**
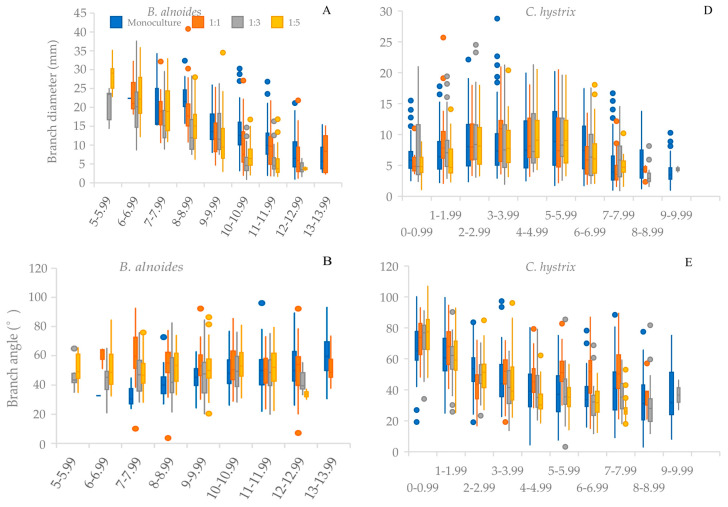

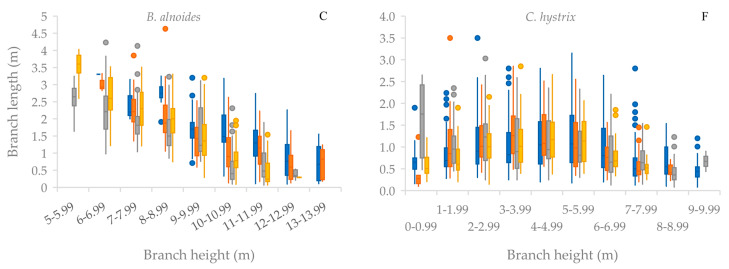
Branch diameter, length and angle variation in different crown layers along the stem for *Betula alnoides,* and *Castanopsis hystrix* in their monocultures and mixtures with different ratios. The edges of the box are 25th and 75th percentiles; the vertical lines are drawn from the box to the most extreme point within 1.5 interquartile ranges; the transverse lines in boxes connect the medians. (**A**) Branch diameter of *B. alnoides*; (**B**) Branch angle of *B. alnoides*; (**C**) Branch length of *B. alnoides*; (**D**) Branch diameter of *C. hystrix*; (**E**) Branch angle of *C. hystrix*; (**F**) Branch length of *C. hystrix*.

The branch diameter and length of *B. alnoides* showed an obvious decreasing trend with increasing crown height (Figure 3A,C). For *C. hystrix*, these traits increased to a peak in the middle crown layer (4–5.99 m) and then decreased (Figure 3D,F). The branch angle of both species presented totally different trends: *B. alnoides* showed a slight increase (Figure 3B), whereas *C. hystrix* exhibited an obvious decrease, with extremely acute angles occurring in the top crown (Figure 3E). Mixing treatments had little effect on vertical variation in branch morphology, except that branch size of *B. alnoides* in most crown layers was substantially reduced in the 1:3 mixture (Figure 3).

Branch distribution across the four cardinal directions was not significantly affected by mixing for both species, regardless of branches in the stem below or above 10 m for *B. alnoides*, and dead or live branches for *C. hystrix* (Table 4). A significant exception was found for *B. alnoides* in the 1:1 mixture, where southward and northward branches significantly outnumbered eastward and westward ones.

## 3. Discussion

### 3.1. Branch Quantity

In the present study, branch number and density in the lower crown section (below 10 m) of *Betula alnoides* were significantly higher in mixtures than in monoculture, while far more branches were observed in the upper crown (above 10 m) under monoculture. In contrast, mixing had no marked effect on the number and density of live or dead branches for *Castanopsis hystrix*. These patterns can be attributed to interspecific growth differences. *B. alnoides* grows faster than *C. hystrix*, and their mixture forms a structurally complementary canopy [25,26], which reduces competitive intensity, and increases light availability in the lower crown of *B. alnoides*, thereby lowering natural pruning intensity. Previous studies have reported that branch number is negatively correlated with competitive intensity [27,28], and positively correlated with light availability [16,29]. This interpretation could be further supported by the lack of significant difference between the monoculture and the 1:1 mixture, and increased number of branches in the 1:3 and 1:5 treatments (Table 2), where intraspecific competition for *B. alnoides* was much higher in monoculture and the 1:1 mixture than in the 1:3 and 1:5 mixtures. The greater number of branches in the upper crown (above 10 m) of *B. alnoides* in monoculture is mainly due to its greater tree height (Table 5), while branch density in this section did not differ significantly among treatments (Table 2), as branches in this crown section experience limited competition.

For *C. hystrix*, although the number of live and dead branches showed no significant difference across all treatments; the total number of branches was markedly higher in monoculture than in mixtures. This result is also correlated with the greater tree height of *C. hystrix* in monoculture (Table 1). Increased intraspecific competition in monoculture promotes height growth, leading to a greater total number of branches [30,31]. Moreover, *C. hystrix* is shade-tolerant [24], and exhibits weak natural pruning and branch shedding during early stand development, which further explains the observed results. The absence of significant differences in branch density among all treatments for *C. hystrix* and in the upper crown of *B. alnoides* indicated that branch emergence may be primarily under genetic control [32,33].

### 3.2. Branch Morphology

Given the strong positive correlation between branch diameter and length [8], both traits responded similarly to mixing treatments in the present study. Mixing had no significant effect on the mean branch size for both *B. alnoides* and *C. hystrix*. This partially contradicts previous studies on young *B. alnoides* mixed with *Erythrophleum fordii* and *Pinus kesiya* [16], as well as on mid-aged mixtures of *Populus tremuloides* and *Picea glauca* [34], where mean branch size was significantly influenced by mixing. Juchheim (2017) also reported that the branch length and diameter of *Fagus sylvatica* were significantly smaller in mixed plantations than in monoculture [35]. These discrepancies may be attributed to differences in mixed species composition, mixing pattern, and stand age. However, branches of *B. alnoides* in the crown section of above 10 m were notably larger in monoculture than in mixtures. One possible explanation is that fewer branches were left in the crown section of below 10 m, allowing more resources to be allocated to these branches for development [18,19,36,37,38]. Meanwhile, mixing with *C. hystrix* reduced competition pressure on *B. alnoides*, especially for branches in the lower crown section, resulting in a larger maximum branch (Table 4) and more large branches (Figure 1). For *C. hystrix*, owing to its shade tolerance and low natural pruning capacity, branch size did not differ significantly among the treatments.

Branch angle is largely genetically determined and generally stable [11,39]. This could be demonstrated through the insignificant differences in branch angle among treatments for *B. alnoides* in the crown section of above 10 m and for *C. hystrix* (Table 4). Nevertheless, this stability is not absolute. Branch angle can vary with branch age (status), diameter [40], and the intensity of external competition [7,41], reflecting the combined effects of multiple factors. In the crown section below 10 m, the branch angle of *B. alnoides* was significantly larger in the mixed stand, especially in the 1:1 mixture, than in monoculture. This may be due to the canopy complementary of the two species, where reduced horizontal competition for space allowed for the development of more horizontal branches. On the contrary, Bayer et al. (2013) found that branch angle of *Fagus sylvatica* was significantly larger in monocultures than in mixtures [18]. These inconsistencies highlight the high plasticity and species-specific nature of branch angle.

### 3.3. Branch Spatial Distribution

Horizontally, branches of both species were evenly distributed across the four cardinal directions and were generally independent of mixing treatments. The only exception occurred for *B. alnoides* in the 1:1 mixture, where branch number was significantly higher in the southern and northern directions than in the eastern and western directions. This asymmetry likely resulted from the fact that *C. hystrix* was planted in east–west-oriented rows, leading to weaker competition in the north–south direction. Additionally, the 2 m (east–west) × 3 m (north–south) planting spacing further intensified competition and mechanical friction, thereby increasing natural pruning [42].

Vertically, branches of both species were predominantly distributed in the upper crown layers across all treatments, consistent with previous studies on *B. alnoides* in mixed plantations [16]. This pattern reflects stronger natural pruning in the lower crown layers, and a reduced competition pressure for *B. alnoides* with the increasing proportions of *C. hystrix*, which shifted the peak branch density upward as the mixing ratio of *C. hystrix* decreased (Figure 2A). For *C. hystrix*, increasing the mixing ratio intensified intraspecific competition, but the vertical distribution of branches was less responsive to mixing than for *B. alnoides* due to its shade tolerance and low natural pruning ability. This is reflected by the vertical profile of branch number (Figure 2B). However, the number of dead branches increased with the proportion of *C. hystrix* (Figure 2D).

Different from the vertical distribution of branch number, the branch diameter and length of *B. alnoides* decreased with increasing crown layer height. This trend is mainly due to the fact that small branches pruned more easily [8,22], leaving only large branches in the lower crown layers. This also explains the much higher variation observed in monoculture, where competition was the most intense. Furthermore, branches in the upper crown layers emerged later than those in the lower crown layers, reinforcing the observed vertical variation pattern. For *C. hystrix*, due to the species-specific attributes mentioned above, branch size varied less vertically, peaking in the middle of crown layers, where light conditions were favorable and branches had a longer growth period than in the upper crown layers. Branch angle also exhibited species-specific vertical variation trends. *C. hystrix* developed more acute angles in the top crown, a branching strategy to alleviate vertical competition in lower crown layers, whereas *B. alnoides* showed less variation with height; this can be attributed to the species-specific attributes of branching.

## 4. Materials and Methods

### 4.1. Study Site

The mixed-species trial plantation was established in March 2017, in Pingxiang City, Guangxi Zhuang Autonomous Region, China (22°02′ N, 106°52′ E; altitude 420 m). This site experiences a northern tropical monsoon climate. The mean annual temperature is approximately 22 °C, with recorded extremes of −1.5 °C and 40.3 °C. The mean annual precipitation is 1550 mm, concentrated primarily during the rainy season from May to September. The mean annual sunshine duration is 1614 h. The soil is yellow-red soil developed from siliceous rock, with organic matter content of 22.43 g·kg^−1^, total N of 0.94 g·kg^−1^, total P of 0.47 g·kg^−1^, total K of 4.6 g·kg^−1^, available N of 117.0 mg·kg^−1^, available P of 1.1 mg·kg^−1^, available K of 85.1 mg·kg^−1^, and a pH of 4.5.

### 4.2. Experimental Design

A randomized complete block design with three replicates was employed for this experiment. The treatments included monocultures of *Betula alnoides* and *Castanopsis hystrix*, as well as three mixtures of the two species at ratios of 1:1, 1:3, and 1:5 (*B. alnoides*–*C. hystrix*). Two blocks were situated on the lower slope, and one on the upper slope. The experimental plantation was established at a spacing of 2 m × 3 m using *B. alnoides* clonal plantlets and *C. hystrix* seedlings, which were cultivated at the Experimental Center of Tropical Forestry, Chinese Academy of Forestry. Each plot covered an area of approximately 0.4 hectares.

### 4.3. Branch Measurement

In April 2023, a subplot (30 m × 30 m) was established at the center of each plot to avoid edge effects. Within these subplots, tree height (H), diameter at breast height (DBH), height to crown base (HCB), and crown diameter (CW) were measured for all trees. H, HCB and CW were measured using a telemeter rod (precision 0.1 m), and DBH was measured with a steel diameter tape (precision 0.1 cm). For branch analysis, three to six dominant or co-dominant trees per species were selected within each treatment, and a total of 40 (20 *B. alnoides* and 20 *C. hystrix*) trees were sampled. The sampled trees should have complete crowns, straight trunks, and be free from insect damage and disease. Additionally, they should be surrounded by healthy trees in all directions. The growth performance of these sampled trees is summarized in Table 5.

All branches within the crown of each sampled tree were measured sequentially from the bottom upwards. For each branch (live or dead), length (m), diameter (mm), insertion angle (°), compass orientation, and height (m) from the ground were measured. Dead branches were defined as those without green leaves and with signs of desiccation, or incompletely occluded branch stubs. Branch diameter (over bark) was measured at the base using an electronic Vernier caliper (0.01 mm) (Guanglu, Guilin, China). Branch length was defined as the distance from the branch–stem junction to the branch tip, and branch height was the vertical distance from the branch–stem junction to the ground; both were all measured using a telemeter rod (0.01 m) (Geelii, Shanghai, China). Branch insertion angle, defined as the angle between the branch axis and the upper stem, was measured with an electronic protractor (1°) (Syntek, Zhuhai, China). Branch orientation was categorized into four cardinal quadrants: east, south, west, and north. All trees were felled to enable in situ measurement of all branches. A total of 3216 branches were measured from 40 trees. Branch density was calculated as the mean number of branches per meter along the stem within the crown.

### 4.4. Data Analysis

Given that the maximum tree height of *C. hystrix* reached up to 10 m, the crown of *B. alnoides* was stratified into two sections for analysis: below and above 10 m. Due to the hierarchical nature of the branch trait data (branches nested within trees, trees nested within plots), linear mixed-effects models (LMMs) were used to test the significance of differences in branch quantitative and morphological attributes among monoculture and mixtures, with different mixing ratios at both the tree and branch levels. The models included mixing treatment, crown layer, and branch orientation as fixed effects, with block, plot, and tree incorporated as random effects. The model was specified as

y_bpt_ = μ + β_M_ + β_C_ + β_O_ + μ_b_ + μ_bp_ + μ_bpt_ + η

where y represents the dependent variable; μ is the overall mean; β_M_ denotes the fixed effect of mixing treatment, β_C_ represents the fixed effect of crown layer; β_O_ represents the fixed effect of branch orientation; μ_b_, μ_bp_, and μ_bpt_ correspond to the random effects for block (b), plot (p), and tree (t), respectively; and η denotes the residual error. Restricted maximum likelihood (REML) estimation was employed in analysis for these models. For fixed effects showing significance, multiple range tests between treatments were conducted using least-square means with Bonferroni adjustment. All analyses were performed in SPSS 21.0 for Windows (IBM-SPSS Inc. Chicago, IL, USA).

## 5. Conclusions

This study evaluated the branch quantity, morphology, and distribution of *Betula alnoides* and *Castanopsis hystrix* in their monocultures and mixtures with different ratios. Mixing significantly modulated branch morphology and quantity for *B. alnoides*, with the response magnitude being both crown layer- and mixing ratio-dependent. Increasing the mixing ratio of *C. hystrix* increased both the number and density of lower-crown branches and the frequency of large branches (>2.5 cm) for *B. alnoides*, while without compromising branch size and quantity in *C. hystrix*. Branch azimuthal distribution remained radially uniform for both species across all treatments, while vertical branch differentiation was primarily governed by inherent species architecture rather than mixing regime. These results demonstrate that mixing ratios can be strategically leveraged to manipulate branch development. However, it could be concluded that the present mixing pattern of *B. alnoides* with *C. hystrix* should be optimized to fully achieve the goal of high-quality solid wood production. Furthermore, long-term studies are needed to evaluate the continued effects of mixing regimes on knot development and tree growth over time. Future study should also explore alternative mixing patterns, as well as correlations between branch development and stem quality, to optimize silvicultural strategies for timber production.

## Figures and Tables

**Table 1:** Branch number statistics of *Betula alnoides* and *Castanopsis hystrix* under monoculture and mixtures with different ratios.

Tree Species	Mixing Ratio	BN			BP		BY		
		Live	Dead	Total	Live	Dead	Live	Dead	Total
*B. alnoides* (<10 m)	Mono.	8.40 (1.47) c	0 (0)	8.40 (1.47) c	1 (0)	0 (0)	3.72 (0.84) c	0 (0)	3.72 (0.84) c
	Mix. (1:1)	18.83 (3.23) bc	0 (0)	18.83 (3.23) bc	1 (0)	0 (0)	6.03 (0.56) ab	0 (0)	6.03 (0.56) ab
	Mix. (1:3)	35.83 (4.28) a	0 (0)	35.83 (4.28) a	1 (0)	0 (0)	7.80 (0.51) a	0 (0)	7.80 (0.51) a
	Mix. (1:5)	28.67 (4.39) ab	0 (0)	28.67 (4.39) ab	1 (0)	0 (0)	5.70 (0.66) b	0 (0)	5.70 (0.66) b
*B. alnoides* (≥10 m)	Mono.	46.22 (6.34) a	0 (0)	46.22 (6.34) a	1 (0)	0 (0)	13.73 (1.56) a	0 (0)	13.73 (1.56) a
	Mix. (1:1)	35.67 (4.96) ab	0 (0)	35.67 (4.96) ab	1 (0)	0 (0)	15.11 (1.85) a	0 (0)	15.11 (1.85) a
	Mix. (1:3)	30.83 (6.48) b	0 (0)	30.83 (6.48) b	1 (0)	0 (0)	17.17 (0.67) a	0 (0)	17.17 (0.67) a
	Mix. (1:5)	32.16 (3.12) ab	0 (0)	32.16 (3.12) ab	1 (0)	0 (0)	18.04 (1.47) a	0 (0)	18.04 (1.47) a
*C. hystrix*	Mono.	78.60 (7.19) a	49.40 (5.85) a	128.00 (7.56) a	0.63 (1.22) a	0.37 (0.45) a	8.28 (1.18) a	4.83 (0.73) a	13.12 (1.22) a
	Mix. (1:1)	65.33 (6.93) a	26.00 (3.51) a	91.33 (10.08) b	0.72 (1.48) a	0.28 (0.15) a	8.65 (1.07) a	3.43 (0.46) a	12.09 (1.49) a
	Mix. (1:3)	62.00 (1.00) a	38.00 (4.00) a	100.00 (3.00) b	0.69 (1.21) a	0.31 (0.69) a	8.81 (0.89) a	4.11 (1.14) a	12.92 (1.21) a
	Mix. (1:5)	55.33 (3.48) a	34.33 (8.65) a	89.67 (11.06) b	0.63 (1.13) a	0.37 (0.63) a	7.11 (0.06) a	4.34 (1.19) a	11.45 (1.33) a

BN: branch number, the number of branches for each tree; BP: branch proportion, the ratio of live/dead branch number and total branch number; BY: branch density was calculated as the mean number of branches per meter along the stem within the crown. Figures in parentheses are the standard error of the mean values, and values followed by different letters show significant differences (*p* < 0.05) between treatments within species.

**Table 2:** F-values and significance levels for mixing ratio (MR), branch orientation (BO), crown layer (CL) and their interactions on the branch morphology of *Betula alnoides* and *Castanopsis hystrix*.

Factors	*B. alnoides*			*C. hystrix*		
	BA	BL	BD	BA	BL	BD
MR	1.087	1.794	2.613	1.073	0.307	0.181
CL	3.060 **	31.502 **	21.149 **	52.059 **	21.380 **	17.419 **
BO	2.151	2.997 *	3.423 *	2.504	1.856	1.098
MR × CL	2.253 **	2.171 **	1.319	4.002 **	3.377 **	1.464
MR × BO	0.839	3.138 **	1.700	2.129 *	1.864	1.778
CL × BO	1.285	1.617 *	1.211	1.137	0.967	0.888
MR × CL × BO	1.326	1.806 **	1.407 *	1.159 *	0.943	0.965

BA, branch angle (°); BL, branch length (m); and BD, branch diameter (mm). **, *p* < 0.01 and *, *p* < 0.05.

**Table 3:** Summary statistics of the branch morphological attributes for *Betula alnoides* and *Castanopsis hystrix* in their monoculture and mixtures with different ratios.

Tree Species	Mixing Ratio	BA (°)	BD (mm)	BL (m)	DLB (mm)	HLB (m)
*B. alnoides* (<10 m)	Mono.	41.02 (4.16) b	19.12 (1.88) a	2.32 (0.29) a	25.39 (3.11) b	8.66 (0.69) a
	Mix. (1:1)	52.28 (3.32) a	18.91 (1.49) a	2.24 (0.23) a	31.93 (2.84) ab	7.55 (0.79) a
	Mix. (1:3)	46.28 (3.14) ab	15.76 (1.40) a	1.89 (0.21) a	32.40 (2.31) ab	6.73 (0.43) a
	Mix. (1:5)	49.00 (3.20) ab	20.36 (1.43) a	2.65 (0.22) a	32.80 (0.91) a	7.15 (0.59) a
*B. alnoides* (≥10 m)	Mono.	52.69 (1.99) a	9.53 (1.49) a	1.02 (0.21) a	/	/
	Mix. (1:1)	50.51 (2.01) a	8.10 (1.43) ab	0.81 (0.20) ab	/	/
	Mix. (1:3)	47.43 (2.28) a	4.20 (1.53) b	0.38 (0.21) b	/	/
	Mix. (1:5)	52.19 (2.24) a	6.19 (1.46) ab	0.67 (0.20) ab	/	/
*C. hystrix*	Mono.	43.40 (2.67) a	7.01 (0.54) a	0.85 (0.07) a	26.67 (3.73) a	4.54 (1.47) a
	Mix. (1:1)	51.12 (3.68) a	7.76 (0.78) a	0.96 (0.10) a	22.37 (1.67) a	3.49 (2.42) a
	Mix. (1:3)	42.73 (3.64) a	7.58 (0.76) a	0.97 (0.10) a	23.04 (0.94) a	2.96 (1.02) a
	Mix. (1:5)	43.83 (3.67) a	7.16 (0.77) a	0.89 (0.10) a	19.23 (1.24) a	3.18 (1.05) a

BA, branch angle (°); BD, branch diameter (mm); BL, branch length (m); DLB, diameter of the largest branch (mm); and HLB, height of the largest branch (m). Figures in parentheses are the standard error of the mean values, and the values followed by different letters indicate significant differences (*p* < 0.05) between treatments within species.

**Table 4:** Branch distribution at the four orientations for *Betula alnoides*, and *Castanopsis hystrix* in their monocultures and mixtures with different ratios.

Tree Species	Mixing Ratio	Branch I				Branch II				Total			
		North	South	East	West	North	South	East	West	North	South	East	West
*B. alnoides*	Mono.	2.00 (1.41) Ba	3.00 (1.41) Ba	1.80 (0.84) Ba	1.60 (1.14) Ba	13.00 (1.30) Aa	13.40 (2.50) Aa	8.60 (1.89) Aa	13.40 (2.50) Aa	15.00 (1.05) Aa	16.40 (2.94) Aa	10.40 (2.04) Aa	12.80 (2.13) Aa
	Mix. (1:1)	6.00 (1.06) Aa	5.83 (1.51) Ba	4.20 (0.80) Ba	3.50 (0.56) Ba	10.67 (1.41) ABa	10.50 (1.23) ABa	7.67 (1.02) ABa	6.83 (1.49) ABa	16.67 (1.86) Aa	16.33 (1.37) Aa	11.17 (1.83) Ab	10.33 (3.89) Ab
	Mix. (1:3)	9.16 (0.91) Aa	9.67 (1.48) Aa	9.00 (1.65) Aa	8.00 (1.15) Aa	6.67 (2.70) Ba	6.00 (2.11) Ba	4.33 (1.15) Ba	4.37 (1.25) Ba	15.83 (2.18) Aa	15.67 (2.73) Aa	13.33 (1.38) Aa	12.83 (1.62) Aa
	Mix. (1:5)	8.17 (1.47) Aa	9.50 (1.59) Aa	6.00 (1.63) Aa	5.00 (0.97) Aa	9.00 (0.73) ABa	8.33 (1.28) ABa	6.67 (0.61) ABa	8.17 (1.22) ABa	17.17 (1.33) Aa	17.83 (2.09) Aa	12.67 (1.78) Aa	12.17 (1.35) Aa
*C. hystrix*	Mono.	22.67 (3.25) Aa	21.33 (3.11) Aa	15.33 (2.44) Aa	14.17 (3.40) Aa	10.00 (2.96) Aa	9.83 (2.30) Aa	15.67 (1.20) Aa	10.17 (1.78) Aa	32.67 (5.68) Aa	31.17 (4.58) Aa	31.00 (2.66) Aa	24.33 (3.86) Aa
	Mix. (1:1)	20.67 (2.91) Aa	18.33 (3.28) Aa	13.00 (2.66) Aa	13.33 (1.86) Aa	7.00 (0.58) Aa	7.00 (1.00) Aa	5.67 (1.76) Ba	6.33 (0.88) Aa	27.67 (3.48) Aa	25.33 (4.26) Aa	18.67 (3.84) Ba	19.67 (1.20) Aa
	Mix. (1:3)	17.67 (3.48) Aa	21.00 (5.29) Aa	15.33 (3.84) Aa	19.00 (3.06) Aa	7.67 (0.67) Aa	10.33 (2.91) Aa	6.33 (1.45) ABa	8.00 (1.73) Aa	25.33 (3.48) Aa	31.33 (2.40) Aa	21.67 (2.73) ABa	27.00 (4.51) Aa
	Mix. (1:5)	13.00 (2.08) Aa	16.33 (1.86) Aa	12.33 (0.88) Aa	13.67 (1.20) Aa	7.67 (2.60) Aa	10.33 (2.40) Aa	10.33 (2.02) ABa	6.00 (2.08) Aa	20.67 (3.28) Aa	26.67 (2.07) Aa	22.67 (1.67) ABa	19.67 (1.20) Aa

Branch I and II mean live branches in crown sections below and above 10 m for *B. alnoides*, and live and dead branches for *C. hystrix*, respectively. Figures in parentheses are the standard error of the mean values, and for each tree species, different capital letters in the same column indicate significant differences (*p* < 0.05) between mixing treatments within species, and different lowercase letters in the same row indicate significant differences between four orientations within the same treatment.

**Table 5:** Growth performance of sampled dominant and codominant trees of *Betula alnoides*, and *Castanopsis hystrix* in their monoculture and mixtures with different ratios.

Tree Species	Mixing Ratio	DBH (cm)	H (m)	HCB (m)	CL(m)	CW (m)
*B. alnoides*	Mono.	11.32 (0.89) a	13.90 (0.52) a	8.18 (0.45) a	5.72 (0.82) a	4.86 (0.39) b
	Mix. (1:1)	9.73 (0.43) a	12.36 (0.38) b	6.31 (0.63) b	6.04 (0.40) a	5.55 (0.27) ab
	Mix. (1:3)	10.41 (0.58) a	11.30 (0.45) b	4.59 (0.55) c	6.71 (0.53) a	5.90 (0.49) ab
	Mix. (1:5)	11.16 (0.43) a	11.85 (0.16) b	4.79 (0.32) c	7.06 (0.28) a	6.45 (0.36) a
*C. hystrix*	Mono.	7.34 (0.49) a	9.64 (0.49) a	0.26 (0.04) b	2.30 (0.33) a	4.69 (0.14) a
	Mix. (1:1)	6.27 (0.20) a	8.50 (0.20) a	0.37 (0.09) a	2.23 (0.40) a	4.58 (0.32) a
	Mix. (1:3)	6.87 (0.13) a	8.48 (0.13) a	0.24 (0.03) b	1.61 (0.63) a	4.84 (0.12) a
	Mix. (1:5)	6.77 (0.23) a	8.14 (0.23) a	0.27 (0.07) b	1.91 (0.10) a	4.19 (0.25) a

DBH: diameter at breast height (cm); H: tree height (m); HCB: height to crown base (m); CL: crown length (m); and CW: crown width (m). The figures in parentheses are the standard error of the mean value, and values followed by different letters show a significant difference (*p* < 0.05) between treatments within species.

## Data Availability

The data are available within the article.

## References

[1] Ishii H.T., Tanabe S.I., Hiura T. (2004). Exploring the Relationships Among Canopy Structure, Stand Productivity, and Biodiversity of Temperate Forest Ecosystems. For. Sci..

[2] Weiskittel A.R., Seymour R.S., Hofmeyer P.V., Kershaw J.A. (2010). Modelling primary branch frequency and size for five conifer species in Maine, USA. For. Ecol. Manag..

[3] Wang C.S., Zhao Z.G., Hein S., Zeng J., Schuler J., Guo J.J., Guo W.F., Zeng J. (2015). Effect of Planting Density on Knot Attributes and Branch Occlusion of *Betula alnoides* under Natural Pruning in Southern China. Forests.

[4] Höwler K., Vor T., Seidel D., Annighöfer P., Ammer C. (2019). Analyzing effects of intra- and interspecific competition on timber quality attributes of *Fagus sylvatica* L.—From quality assessments on standing trees to sawn boards. Eur. J. For. Res..

[5] Stapel P., Kuilen J.W.G.V.D. (2014). Influence of cross-section and knot assessment on the strength of visually graded Norway spruce. Eur. J. Wood Wood Prod..

[6] Collins S., Fink G. (2022). Modeling the tensile mechanical properties of silver birch timber boards. Constr. Build. Mater..

[7] Alcorn P.J., Pyttel P., Bauhus J., Smith R.G.B., Thomas D., James R., Nicotra A. (2007). Effects of initial planting density on branch development in 4-year-old plantation grown *Eucalyptus pilularis* and *Eucalyptus cloeziana* trees. For. Ecol. Manag..

[8] Wang C.S., Tang C., Hein S., Guo J.J., Zhao Z.G., Zeng J. (2018). Branch development of five-year-old *Betula alnoides* plantations in response to planting density. Forests.

[9] Zhang Y., Chen H.Y.H., Reich P.B. (2012). Forest productivity increases with evenness, species richness and trait variation: A global meta-analysis. J. Ecol..

[10] Jucker T., Bouriaud O., Avacaritei D., Coomes D.A. (2014). Stabilizing effects of diversity on aboveground wood production in forest ecosystems: Linking patterns and processes. Ecol. Lett..

[11] Lowell E.C., Maguire D.A., Briggs D.G., Turnblom E.C., Jayawickrama K.J., Bryce J. (2014). Effects of Silviculture and Genetics on Branch/Knot Attributes of Coastal Pacific Northwest Douglas-Fir and Implications for Wood Quality—A Synthesis. Forests.

[12] Santos F.M., Chaer G.M., Diniz A.R., Balieiro F.D.C. (2017). Nutrient cycling over five years of mixed-species plantations of Eucalyptus and Acacia on a sandy tropical soil. For. Ecol. Manag..

[13] Lisella C., Bottero A., Antonucci S., Santopuoli G., Tognetti R. (2025). Resilience to late frost and drought of mixed forests with Turkey oak and silver fir in southern Italy. For. Ecol. Manag..

[14] Cao J., Liu H., Zhao B., Peng R., Liang B., Anenkhonov O.A., Korolyuk A.Y., Sandanov D.V. (2022). Mixed forest suffered less drought stress than pure forest in southern Siberia. Agric. For. Meteorol..

[15] Höwler K., Vor T., Schall P., Annighöfer P., Seidel D., Ammer C. (2021). Distribution of the timber quality attribute ‘knot surface’ in logs of *Fagus sylvatica* L. from pure and mixed forest stands. Eur. J. For. Res..

[16] Liu K.L., Wang C.S., Chen B.Y., Wang R.H., Zeng J. (2023). Branch development in monoculture and mixed-species plantations of *Betula alnoides*, *Erythrophleum fordii* and *Pinus kesiya* var. *langbianensis* in southwestern China. For. Ecol. Manag..

[17] Pretzsch H. (2014). Canopy space filling and tree crown morphology in mixed-species stands compared with monocultures. For. Ecol. Manag..

[18] Bayer D., Seifert S., Pretzsch H. (2013). Structural crown properties of Norway spruce (*Picea abies* [L.] Karst.) and European beech (*Fagus sylvatica* [L.]) in mixed versus pure stands revealed by terrestrial laser scanning. Trees.

[19] Jucker T., Bouriaud O., Coomes D.A., Baltzer J. (2015). Crown plasticity enables trees to optimize canopy packing in mixed-species forests. Funct. Ecol..

[20] Bauhus J., Forrester D.I., Pretzsch H., Felton A., Pyttel P., Benneter A., Pretzsch H., Forrester D.I., Bauhus J. (2017). Silvicultural Options for Mixed-Species Stands in Mixed-Species Forests: Ecology Management.

[21] Jie Z., Wen-Fu G., Zhi-Gang Z., Qi-Jie W., Guang-Tian Y., Hai-Shui Z. (2006). Domestication of *Betula alnoides* in China: Current Status and Perspectives. For. Res..

[22] Wang C.S., Hein S., Zhao Z.G., Guo J.J., Zeng J. (2016). Branch occlusion and discoloration of *Betula alnoides* under artificial and natural pruning. For. Ecol. Manag..

[23] Wang C.S., Guo J.J., Hein S., Wang H., Zhao Z.G., Zeng J. (2019). Foliar morphology and spatial distribution in five-year-old plantations of *Betula alnoides*. For. Ecol. Manag..

[24] Li X., Wu G., Lie Z., Aguila L.C.R., Khan M.S., Luo H., Wu T., Liu X., Liu J. (2025). Microbial community variation in rhizosphere and non-rhizosphere soils of *Castanopsis hystrix* plantations across stand ages. J. For. Res..

[25] Pretzsch H., Schütze G. (2016). Effect of tree species mixing on the size structure, density, and yield of forest stands. Eur. J. For. Res..

[26] Williams L.J., Paquette A., Cavender-Bares J., Messier C., Reich P.B. (2017). Spatial complementarity in tree crowns explains overyielding in species mixtures. Nat. Ecol. Evol..

[27] Cahill J.F., Kembel S.W., Lamb E.G., Keddy P.A. (2008). Does phylogenetic relatedness influence the strength of competition among vascular plants?. Perspect. Plant Ecol. Evol. Syst..

[28] Hildebrand M., Perles-Garcia M.D., Kunz M., Härdtle W., von Oheimb G., Fichtner A. (2021). Tree-tree interactions and crown complementarity: The role of functional diversity and branch traits for canopy packing. Basic Appl. Ecol..

[29] Chen L., Sumida A. (2018). Effects of light on branch growth and death vary at different organization levels of branching units in Sakhalin spruce. Trees.

[30] Muth Christine C., Bazzaz F. (2003). A Tree canopy displacement and neighborhood interactions. Can. J. For. Res..

[31] Lines Emily R., Zavala Miguel A., Purves Drew W., Coomes David A. (2012). Predictable changes in aboveground allometry of trees along gradients of temperature, aridity and competition. Glob. Ecol. Biogeogr..

[32] Mäkinen H., Hein S. (2006). Effect of wide spacing on increment and branch properties of young Norway spruce. Eur. J. For. Res..

[33] Kint V., Hein S., Campioli M., Muys B. (2010). Modelling self-pruning and branch attributes for young *Quercus robur* L. and *Fagus sylvatica* L. trees. For. Ecol. Manag..

[34] Comeau P.G. (2021). Effects of Aspen and Spruce Density on Size and Number of Lower Branches 20 Years after Thinning of Two Boreal Mixedwood Stands. Forests.

[35] Juchheim J., Annighöfer P., Ammer C., Calders K., Raumonen P., Seidel D. (2017). How management intensity and neighborhood composition affect the structure of beech (*Fagus sylvatica* L.) trees. Trees.

[36] Vieilledent G., Courbaud B.T., Kunstler G., Clark J.F.O.D.T.S. (2010). Individual variability in tree allometry determines light resource allocation in forest ecosystems: A hierarchical Bayesian approach. Oecologia.

[37] Pretzsch H., Dieler J. (2012). Evidence of variant intra- and interspecific scaling of tree crown structure and relevance for allometric theory. Springer Open Choice.

[38] Longuetaud F., Piboule A., Wernsdörfer H., Collet C. (2013). Crown plasticity reduces inter-tree competition in a mixed broadleaved forest. Eur. J. For. Res..

[39] West P.W., Smith R.G.B. (2020). Effects of tree spacing on branch-size development during early growth of an experimental plantation of *Eucalyptus pilularis* in subtropical Australia. Aust. For..

[40] Wang C.S., Zeng J., Hein S., Zhao Z.G., Guo J.J., Zeng J. (2017). Crown and branch attributes of mid-aged *Betula alnoides* plantations in response to planting density. Scand. J. For. Res..

[41] Akers M.K., Kane M., Zhao D., Teskey R.O., Daniels R.F. (2013). Effects of planting density and cultural intensity on stand and crown attributes of mid-rotation loblolly pine plantations. For. Ecol. Manag..

[42] Hajek P., Seidel D., Leuschner C. (2015). Mechanical abrasion, and not competition for light, is the dominant canopy interaction in a temperate mixed forest. For. Ecol. Manag..

